# EASIX is an effective marker in predicting mortality of traumatic brain injury patients

**DOI:** 10.1186/s40001-024-01899-0

**Published:** 2024-05-29

**Authors:** Ruoran Wang, Yuelin Jiang, Min He, Jianguo Xu

**Affiliations:** 1 Department of Neurosurgery, West China Hospital, Sichuan University, No. 37, Guoxue Alley, Chengdu, 610041 Sichuan China; 2 West China Clinical Medical College of Sichuan University, Chengdu, Sichuan China; 3 Department of Critical Care Medicine, West China Hospital, Sichuan University, No. 37, Guoxue Alley, Chengdu, 610041 Sichuan China

**Keywords:** Traumatic brain injury, EASIX, Endothelial dysfunction, Mortality, Prediction

## Abstract

**Background:**

The Endothelial Activation and Stress Index (EASIX) is a novel marker of endothelial injury and correlates with survival of various patients. The endothelial dysfunction plays an important role on the pathophysiological process of traumatic brain injury (TBI). This study was designed to explore the prognostic value of EASIX on TBI patients.

**Methods:**

358 TBI patients hospitalized in the West China hospital between October 2018 and October 2022 were enrolled for this study. The EASIX was calculated based on the formula: lactate dehydrogenase (U/L) × creatinine (mg/dL)/platelets (10^9^ cells/L). The univariate and multivariate logistic regression with forward method was performed to explore the association between EASIX and mortality. A prognostic model was developed combining significant risk factors in the multivariate logistic regression. The receiver operating characteristic (ROC) curve was used to compare the predictive accuracy of the EASIX and the developed model.

**Results:**

The 30-day mortality of enrolled 358 TBI patients was 51.1%. Non-survivors had higher EASIX than survivors (*p* < 0.001). The multivariate logistic regression confirmed seven risk factors for mortality of TBI including injury mechanism (*p* = 0.010), GCS (*p* < 0.001), glucose (*p* < 0.001), EASIX (*p* = 0.017), subdural hematoma (*p* = 0.012), coagulopathy (*p* = 0.001). The AUC of EASIX, SOFA, GCS was 0.747, 0.748 and 0.774, respectively. The AUC of developed predictive model was 0.874 with the sensitivity of 0.913 and specificity of 0.686.

**Conclusions:**

The EASIX is a reliable marker for predicting mortality of TBI patients. The predictive model incorporating EASIX is helpful for clinicians to evaluate the mortality risk of TBI patients.

## Introduction

Traumatic brain injury (TBI) is a worldwide disease bringing a huge economic burden to the society and victims. The incidence of TBI is estimated being 69 million per year around the world [1]. Due to the high mortality and poor functional outcome after TBI, many works have been performed to explore prognostic factors and make personalized treatment guidelines for TBI. Many pathophysiological processes take part in the progression of TBI including the endovascular dysfunction. The cerebral microvascular endovascular damage would promote the blood brain barrier (BBB) breakdown and vasogenic edema [2]. In addition, the peripheral terminal endovascular damage prevalent after TBI could promote the platelet adhesion and the microthrombi formation with subsequent coagulopathy, which is associated with higher mortality of TBI [3–6]. Therefore, evaluating the severity of endothelial injury may be helpful for risk stratification of TBI patients.

The endothelial activation and stress index (EASIX) has recently been designed and verified to evaluate the severity of endothelial injury after allogeneic stem-cell transplantation [7]. Researchers found it was associated with levels of endothelial activation markers such as interleukin-18, chemokine-X-C-ligand 8, insulin like-growth-factor-1, suppressor of tumorigenicity-2 [8, 9]. Furthermore, the EASIX has been confirmed related with mortality of various patients such as multiple myeloma, COVID-19, diffuse large B-cell lymphoma, small cell lung cancer, sepsis [10–14]. While the prognostic effect of the EASIX has not been testified in TBI patients, we design this study to analyze the relationship between the EASIX and mortality of TBI.

## Materials and methods

### Patients

TBI patients hospitalized in the West China hospital between October 2018 and October 2022 were selected for this study. Eligible participants were excluded if they met the following criteria: (1) admitted to our hospital 6 h after suffering initial intracranial injury; (2) transferred patients received initial treatments in other hospitals; (3) lacked in relevant variables. 358 TBI patients were finally included. This study was approved by the ethics committee of West China hospital and conducted based on the ethical standards of the Declaration of Helsinki. Informed consent of being enrolled in the observational study of each patient was signed by patients themselves or legally authorized representatives after admission to our hospital.

### Study variables

Demographic variables including age and gender were collected. Injury mechanisms of TBI were classified including traffic accident, high falling, stumbling, others. Vital signs on admission including systolic blood pressure, diastolic blood pressure, heart rate, body temperature were recorded. The severity of TBI was evaluated by Glasgow Coma Scale (GCS), Injury Severity Score (ISS), Sequential Organ Failure Assessment (SOFA). Levels of white blood cell, platelet, hemoglobin, serum creatinine, glucose, lactate dehydrogenase, prothrombin time were obtained by analyzing the first blood sample within the first day after admission. The Endothelial Activation and Stress Index (EASIX) was calculated based on the formula: lactate dehydrogenase (U/L) × creatinine (mg/dL)/platelets (10^9^ cells/L). The intracranial injury was classified including epidural hematoma, subdural hematoma, subarachnoid hemorrhage, delayed axonal injury. The primary outcome of this study was the 30-day mortality. The incidence of coagulopathy, length of ICU stay, and length of hospital stay were compared between survivors and non-survivors.

### Statistical analysis

Through the Kolmogorov–Smirnov test, normality of variables was tested. Non-normal distributed variables and normal distributed variables were presented as median (interquartile range), and mean ± standard deviation, respectively. Mann–Whitney *U* test and Student’s *t* test were used to analyze differences of non-normal distributed variables and normal distributed variables between survivors and non-survivors. The chi-square test or Fisher exact test was used to analyze differences of categorical variables between survivors and non-survivors. Spearman correlation method was conducted to analysis the relationship between EASIX and other variables. The restricted cubic spline (RCS) method was used to generally explore the relationship between EASIX and mortality of TBI. Then, univariate logistic regression was performed to explore potential risk factors of mortality. Those significant factors were then analyzed in the multivariate logistic regression with forward method. A prognostic model was finally developed combining significant risk factors in the multivariate logistic regression. The nomogram of this model was drawn for convenient clinical use. And a calibration plot was also drawn to evaluate the stability of this model. The receiver operating characteristic (ROC) curve was used to compare the predictive accuracy of the EASIX and the developed model. The Delong test was used to compare the difference of the area under the ROC curve (AUC) between the EASIX, GCS, SOFA, and the developed model.

*P* value < 0.05 with two sides was defined as statistically significant. SPSS 23.0 Windows software (SPSS, Inc, Chicago, IL) and R (version 3.6.1; R Foundation) were used for all statistical analyses.

## Results

### Clinical characteristics of included TBI patients

358 TBI patients were enrolled with the 30-day mortality of 51.1% (Table 1). Age (*p* = 0.410) and gender ratio (*p* = 0.246) did not differ between 175 survivors and 183 non-survivors. Non-survivors were more likely to suffer the traffic accident while survivors were more likely to suffer the high falling. The systolic blood pressure (*p* = 0.026) and diastolic blood pressure (*p* = 0.023) were both significantly lower in non-survivors while heart rate (*p* < 0.001) was significantly higher in survivors. Additionally, non-survivors had more severe injury reflected by lower GCS (*p* < 0.001), higher ISS (*p* < 0.001) and higher SOFA (*p* < 0.001). Regarding results of laboratory tests, white blood cell (*p* = 0.021), serum creatinine (*p* < 0.001), glucose (*p* < 0.001), lactate dehydrogenase (*p* < 0.001), prothrombin time (*p* < 0.001) were all significantly higher in non-survivors while platelet (*p* < 0.001) and hemoglobin (*p* < 0.001) were significantly lower in non-survivors. And, the EASIX was significantly higher in non-survivors (*p* < 0.001). The incidence of subdural hematoma (*p* < 0.001), subarachnoid hemorrhage (*p* = 0.029), delayed axonal injury (*p* = 0.020) were all significantly higher in non-survivors. Finally, compared with survivors, non-survivors had higher incidence of coagulopathy (*p* < 0.001), shorter length of ICU stay (*p* < 0.001) and length of hospital stay (*p* < 0.001).

**Table 1:** Baseline information of included TBI patients

Variables	Overall (*n* = 358)	Survivors (*n* = 175, 48.9%)	Non-survivors (*n* = 183, 51.1%)	*p*
Age (year)	46 (33–59)	46 (34–61)	46 (32–58)	0.410
Male gender, n (%)	277 (77.374%)	140 (80.0%)	137 (74.9%)	0.246
Injury mechanism, n (%)				0.004
Traffic accident	226 (63.1%)	96 (54.9%)	130 (71.0%)	
High falling	66 (18.4%)	41 (23.4%)	25 (13.7%)	
Stumbling	39 (10.9%)	19 (10.9%)	20 (10.9%)	
Others	27 (7.5%)	19 (10.9%)	8 (4.4%)	
Admission vital signs				
Systolic blood pressure (mmHg)	122 (107–141)	125 (111–141)	120 (104–142)	0.026
Diastolic blood pressure (mmHg)	74 ± 16	76 ± 13	72 ± 19	0.023
Heart rate (s^−1^)	97 (80–117)	93 (76–109)	103 (87–121)	< 0.001
Body temperature (℃)	36.7 (36.4–37.0)	36.7 (36.5–37.0)	36.7 (36.2–37.0)	0.081
GCS	6 (5–8)	7 (5–11)	5 (4–6)	< 0.001
ISS	25 (16–25)	16 (9–25)	25 (22–25)	< 0.001
SOFA	6 (4–7)	5 (3–6)	7 (5–8)	< 0.001
Admission laboratory data				
White blood cell (10^9^/L)	14.98 (10.78–19.14]	13.87 (10.51–17.89]	15.73 (11.54–20.43]	0.021
Platelet (10^9^/L)	99 (64–154)	126 (84–167)	82 (50–127)	< 0.001
Hemoglobin (g/L)	88 (75–110)	97 (81–117)	83 (72–100)	< 0.001
Serum creatinine (mmol/L)	0.85 (0.67–1.14)	0.77 (0.66–0.94)	0.94 (0.70–1.39)	< 0.001
Glucose (mmol/L)	9.89 (7.48–13.50)	8.47 (6.59–11.24)	12.20 (9.13–15.51)	< 0.001
Lactate dehydrogenase (U/L)	373 (285–536)	319 (243–413)	443 (330–678)	< 0.001
Prothrombin time (s)	13.5 (12.1–15.5)	12.6 (11.8–13.9)	14.6 (13.2–17.1)	< 0.001
EASIX	3.35 (1.55–8.64)	2.03 (1.13–3.68)	5.86 (2.72–14.69)	< 0.001
Intracranial injury classification				
Epidural hematoma, n (%)	36 (10.1%)	18 (10.3%)	18 (9.8%)	0.888
Subdural hematoma, n (%)	99 (27.7%)	32 (18.3%)	67 (36.6%)	< 0.001
Subarachnoid hemorrhage, n (%)	203 (56.7%)	89 (50.9%)	114 (62.3%)	0.029
Delayed axonal injury, n (%)	89 (24.9%)	34 (19.4%)	55 (30.1%)	0.020
Coagulopathy, n (%)	133 (37.2%)	35 (20.0%)	98 (53.6%)	< 0.001
Length of ICU stay (day)	3 (1–18)	14 (0–27)	2 (1–4)	< 0.001
Length of hospital stay (day)	13 (5–29)	27 (16–43)	5 (3–11)	< 0.001

GCS, Glasgow Coma Scale; ISS, Injury Severity Score; SOFA, Sequential Organ Failure Assessment; EASIX, Endothelial Activation and Stress Index

### Association between EASIX and mortality of included TBI patients

**Fig. 1:**
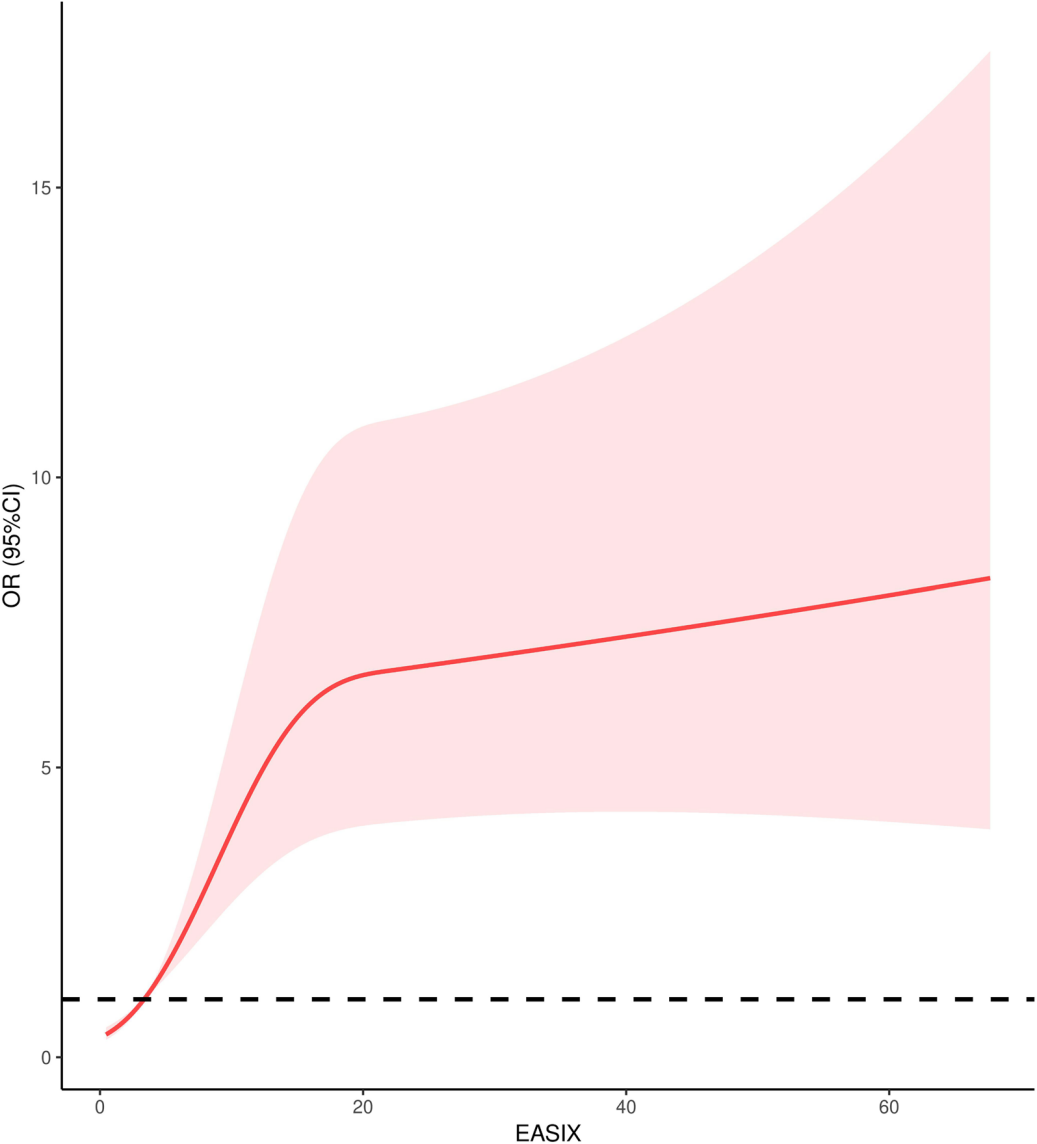
Relationship between the EASIX and mortality of TBI patients

**Fig. 2:**
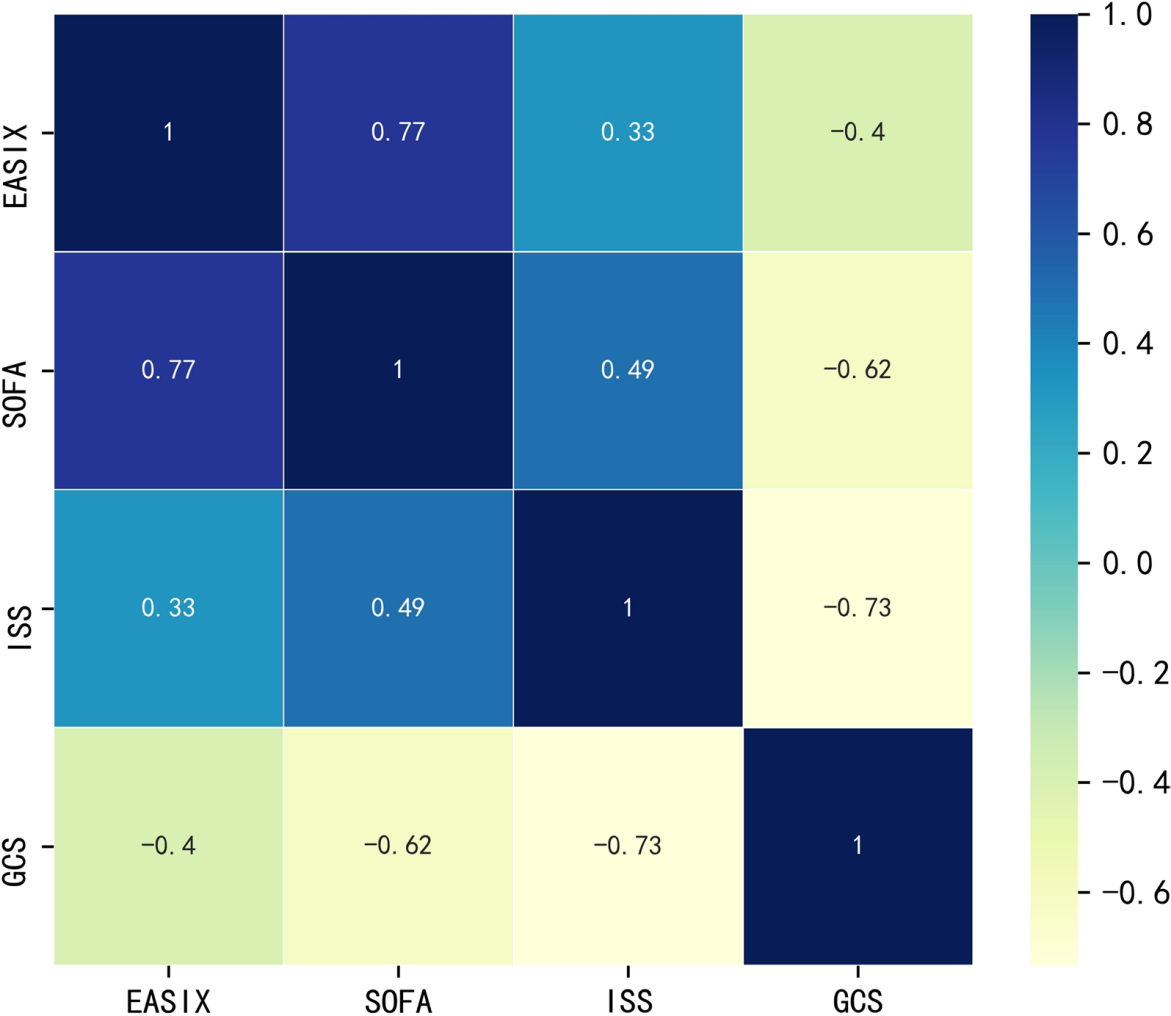
Correlation between the EASIX and the other scores in TBI patients

The RCS showed the EASIX was positively related with the mortality of TBI (Fig. 1). The EASIX was strongly associated with the SOFA score (*r* = 0.771, *p* < 0.001) but mildly associated with the ISS score (*r* = 0.328, *p* < 0.001) and the GCS (*r* = − 0.400, *p* < 0.001) (Fig. 2). The unadjusted logistic regression found injury mechanism (*p* = 0.005), systolic blood pressure (*p* = 0.031), diastolic blood pressure (*p* = 0.026), heart rate (*p* < 0.001), GCS (*p* < 0.001), ISS (*p* < 0.001), SOFA (*p* < 0.001), white blood cell (*p* = 0.027), hemoglobin (*p* < 0.001), glucose (*p* < 0.001), prothrombin time(*p* < 0.001), EASIX (*p* < 0.001), subdural hematoma (*p* < 0.001), subarachnoid hemorrhage (*p* = 0.029), delayed axonal injury (*p* = 0.021), coagulopathy (*p* < 0.001) were related with the mortality (Table 2). However, after adjusting confounded effects, the adjusted logistic regression with forward method confirmed that seven factors were significantly correlated with the mortality of TBI including injury mechanism (*p* = 0.010), GCS (*p* < 0.001), glucose (*p* < 0.001), EASIX (*p* = 0.017), subdural hematoma (*p* = 0.012), coagulopathy (*p* = 0.001).

**Table 2:** Univariate and multivariate logistic regression analysis for exploring risk factors of mortality in TBI patients

	Unadjusted analysis			Adjusted analysis		
	OR	95%CI	P value	OR	95%CI	*P* value
Age	0.995	0.983–1.007	0.424			
Male gender	0.745	0.452–1.226	0.246			
Injury mechanism			0.005			0.010
Traffic accident	1.000	Reference		1.000	Reference	
High falling	0.450	0.256–0.791	0.005	0.406	0.197–0.837	0.015
Stumbling	0.777	0.393–1.536	0.469	2.124	0.858–5.253	0.103
Others	0.311	0.131–0.740	0.008	0.467	0.147–1.483	0.197
Systolic blood pressure	0.991	0.983–0.999	0.031			
Diastolic blood pressure	0.985	0.973–0.998	0.026			
Heart rate	1.017	1.008–1.025	< 0.001			
Body temperature	0.876	0.693–1.108	0.270			
GCS	0.629	0.561–0.705	< 0.001	0.652	0.570–0.746	< 0.001
ISS	1.091	1.063–1.121	< 0.001			
SOFA	1.504	1.341–1.687	< 0.001			
White blood cell	1.036	1.004–1.070	0.027			
Hemoglobin	0.979	0.970–0.988	< 0.001			
Glucose	1.241	1.166–1.320	< 0.001	1.162	1.083–1.246	< 0.001
Prothrombin time	1.355	1.225–1.498	< 0.001			
EASIX	1.067	1.035–1.101	< 0.001	1.022	1.004–1.040	0.017
Epidural hematoma	0.952	0.478–1.895	0.888			
Subdural hematoma	2.581	1.586–4.201	< 0.001	2.222	1.191–4.146	0.012
Subarachnoid hemorrhage	1.596	1.048–2.432	0.029			
Delayed axonal injury	1.782	1.092–2.909	0.021			
Coagulopathy	4.612	2.880–7.384	< 0.001	2.608	1.451–4.689	0.001

GCS, Glasgow Coma Scale; ISS, Injury Severity Score; SOFA, Sequential Organ Failure Assessment; EASIX, Endothelial Activation and Stress Index

### Prognostic value of EAXIS for mortality of TBI patients

**Fig. 3:**
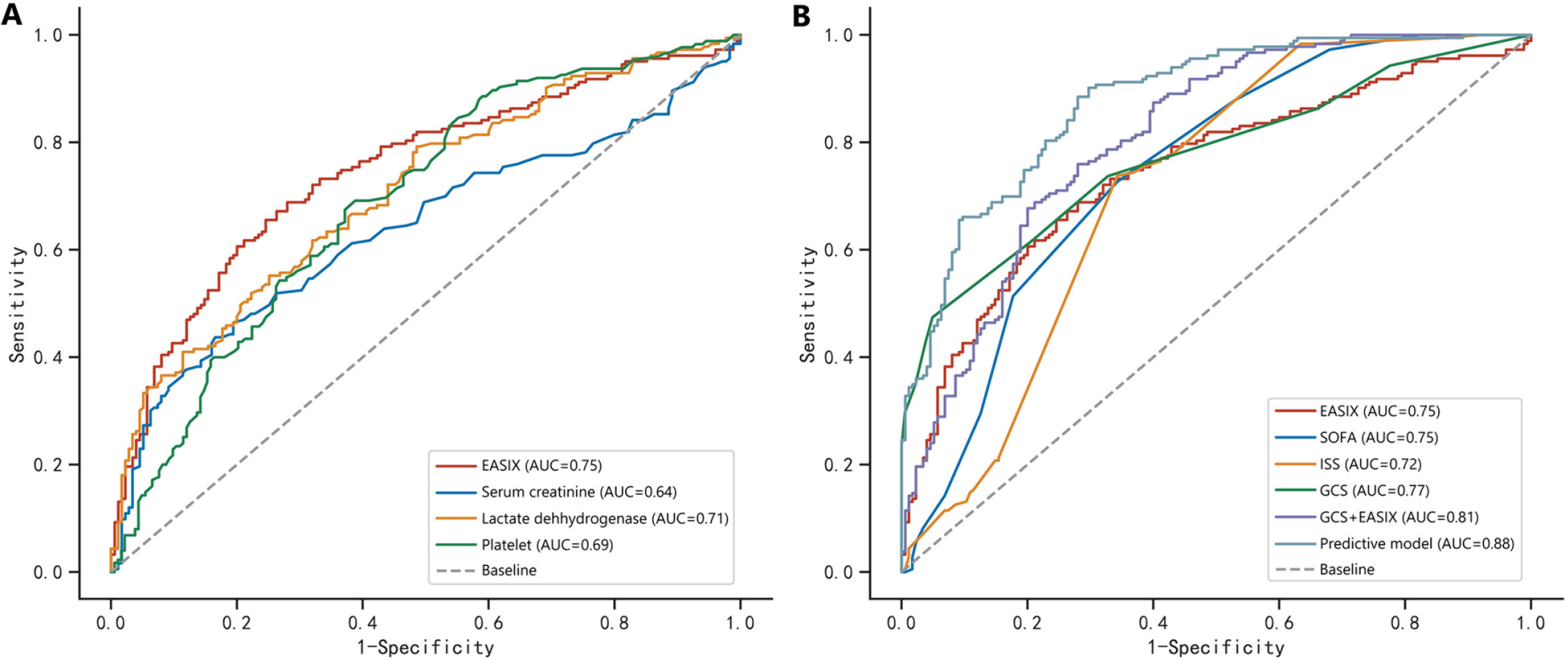
**A** Receiver operating characteristics curve of the EASIX for predicting mortality in TBI patients. **B** Receiver operating characteristics curve of the predictive model for mortality in TBI patients

**Fig. 4:**
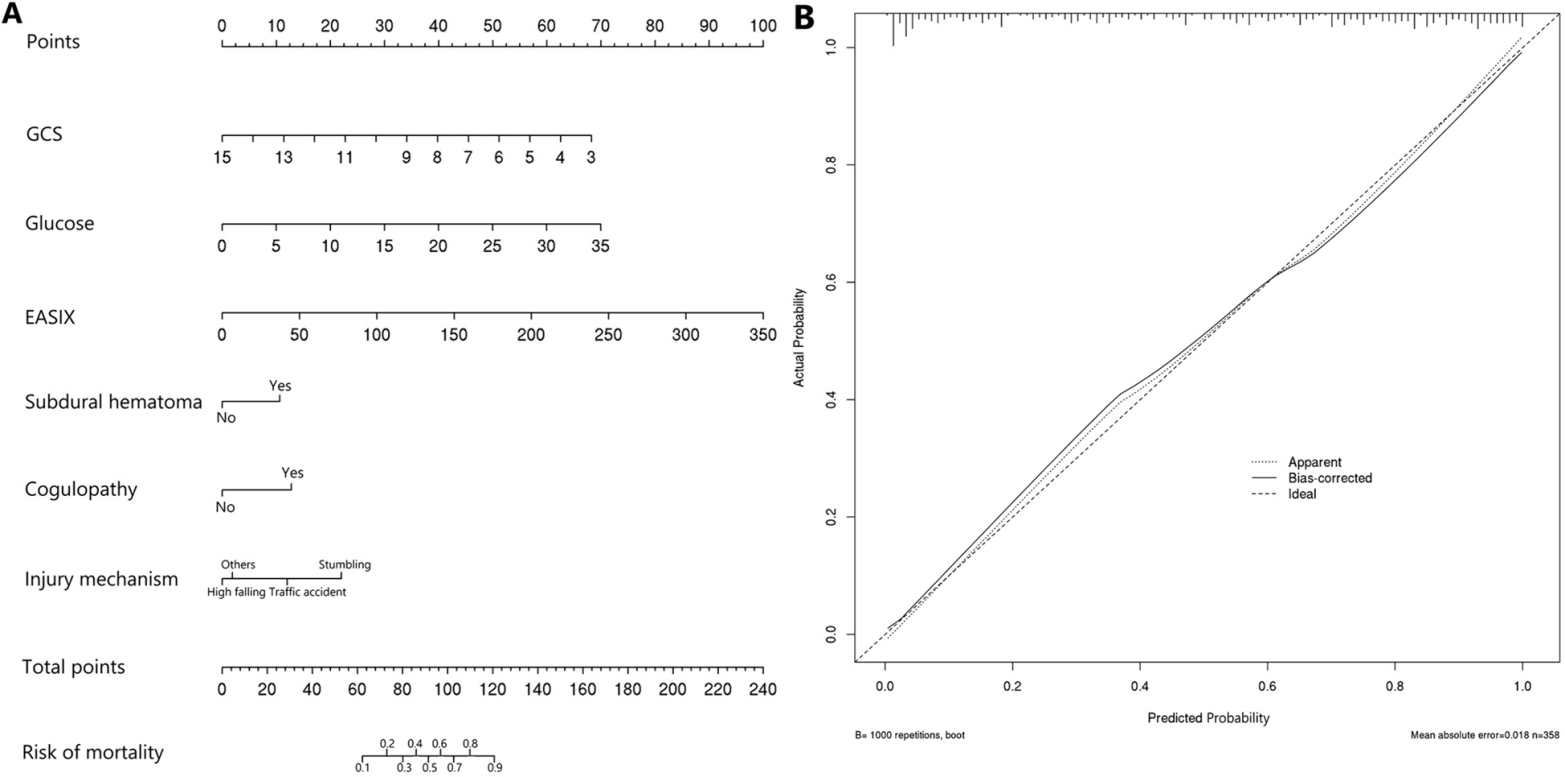
**A** The nomogram of the predictive model for mortality in TBI patients. **B** The calibration plot of the predictive model for mortality in TBI patients

The AUC of single lactate dehydrogenase, serum creatinine, platelet for predicting mortality was 0.709, 0.638, 0.690, respectively (Table 3) (Fig. 3A). Calculating from these three factors, the EASIX had an AUC of 0.747, which was comparable to the 0.748 of SOFA (*Z* = 0.067, *p* = 0.946), 0.716 of ISS (*Z* = 0.921, *p* = 0.357) (Fig. 3B) (Table 4). The AUC of GCS was 0.774, which was relatively higher than that of EASIX (*Z* = 13.073, *p* < 0.001). The AUC of GCS plus EASIX was 0.809, which had been improved comparing with single GCS (*Z* = 12.682, *p* < 0.001) or EASIX (*Z* = 2.513, *p* = 0.012). Finally, the AUC of developed predictive model incorporating GCS, glucose, EASIX, subdural hematoma, injury mechanism, coagulopathy was 0.874 with the sensitivity of 0.913 and specificity of 0.686. The predictive model was visually shown as the nomogram for convenient clinical use (Fig. 4A, B).

**Table 3:** Predictive performance of EASIX and the predictive model for mortality in TBI patients

	AUC	95% CI	Sensitivity	Specificity	Youden index	Cut-off value
SOFA	0.748	0.697–0.799	0.727	0.657	0.384	6
Lactate dehydrogenase	0.709	0.656–0.762	0.792	0.514	0.307	321
Serum creatinine	0.638	0.580–0.696	0.437	0.834	0.271	1.05
Platelet	0.690	0.636–0.745	0.691	0.612	0.303	94
EASIX	0.747	0.696–0.798	0.656	0.754	0.410	3.76
GCS	0.774	0.725–0.822	0.474	0.951	0.425	8
ISS	0.716	0.662–0.771	0.738	0.657	0.395	25
*Coagulopathy*	0.668	0.661–0.724	0.536	0.800	1.336	–
GCS + EASIX	0.809	0.765–0.854	0.76	0.72	0.480	0.53
Predictive model	0.874	0.840–0.909	0.913	0.686	0.599	0.373

GCS, Glasgow Coma Scale; ISS, Injury Severity Score; SOFA, Sequential Organ Failure Assessment; EASIX, Endothelial Activation and Stress Index

The predictive model was composed of GCS, glucose, EASIX, subdural hematoma, injury mechanism, coagulopathy

**Table 4:** Predictive performance comparison between EASIX, SOFA, ISS, GCS, and the predictive model for mortality in TBI patients

	SOFA		ISS		GCS		EASIX		GCS + EASIX		Predictive model	
	*Z*	*p*	*Z*	*p*	*Z*	*p*	*Z*	*p*	*Z*	*p*	*Z*	*p*
SOFA			1.106	0.269	11.869	< 0.001	0.067	0.946	3.094	0.002	5.605	< 0.001
ISS	1.106	0.269			10.372	< 0.001	0.921	0.357	4.172	< 0.001	6.316	< 0.001
GCS	11.869	< 0.001	10.372	< 0.001			13.073	< 0.001	12.682	< 0.001	17.024	< 0.001
EASIX	0.067	0.946	0.921	0.357	13.073	< 0.001			2.513	0.012	5.139	< 0.001
GCS + EASIX	3.094	0.002	4.172	< 0.001	12.682	< 0.001	2.513	0.012			3.831	< 0.001
Predictive model	5.605	< 0.001	6.316	< 0.001	17.024	< 0.001	5.139	< 0.001	3.831	< 0.001		

GCS, Glasgow Coma Scale; ISS, Injury Severity Score; SOFA, Sequential Organ Failure Assessment; EASIX, Endothelial Activation and Stress Index

The predictive model was composed of GCS, glucose, EASIX, subdural hematoma, injury mechanism, coagulopathy

## Discussion

An index named EASIX was primarily designed and confirmed as an effective predictor of overall survival among patients with steroid-refractory graft-versus-host disease after allogeneic stem-cell transplantation which was associated with the thrombotic microangiopathy through endothelial dysfunction [7]. Then, the prognostic value of the EASIX incorporating lactate dehydrogenase, serum creatinine and platelet was verified in many kinds of patients including lower risk myelodysplastic syndromes, multiple myeloma, COVID-19, diffuse large B-cell lymphoma, small cell lung cancer, sepsis, and critically ill patients with advanced liver disease [10–15]. Some studies showed the significant correlation between the EASIX and levels of endothelial activation markers including interleukin-18, chemokine-X-C-ligand 8, C-X-C motif chemokine ligand 9, insulin like-growth-factor-1, suppressor of tumorigenicity-2, angiopoietin-2, soluble thrombomodulin [8, 9]. As the marker of endothelial injury, the EASIX is readily obtained in clinical practice with less costs to evaluate the risk of complication and prognosis.

As the component of EASIX, lactate dehydrogenase would increase due to the release from endothelial cells, platelets and leukocytes when vascular endothelium was damaged [16, 17]. The endothelial dysfunction is a key pathophysiological process of many renal diseases including acute kidney injury, chronic kidney disease, diabetic nephropathy [18–20]. The high serum creatinine level reflects the influence of renal endothelial dysfunction on impairing renal function to a certain degree. Additionally, the low platelet level may also be partly attributable to endothelial injury and complement activation. The increased collagen exposition, von Willebrand factor, and tissue factor caused by the vascular endothelial damage would promote the platelet hyperactivation and hyperaggregation [21]. The level of lactate dehydrogenase, serum creatinine, and platelet has been confirmed associated with the mortality of TBI by previous studies [22–24]. However, as components of the marker reflecting the degree of vascular endothelial injury, their correlation with the prognosis of TBI has not been confirmed.

Our study showed the EASIX was significantly higher in non-survivors and effective in predicting the mortality of TBI. Some underlying mechanisms may be responsible for the association between the EASIX and prognosis of TBI. Firstly, as the critical component of BBB, cerebral microvascular endothelial cell plays an important role on maintaining the integrity of BBB. One recent research indicated that mitochondrial dysfunction of brain microvascular endothelial cells (BMVEC) was a key factor for BBB breakdown and TBI progression [25]. The cerebral microvascular endothelial cell would be damaged by the inflammation, oxidative stress and circulating extracellular vesicles with the subsequent BBB breakdown and vasogenic edema [2, 26]. It has been confirmed that brain edema caused the clinical deterioration in a half of TBI patients [27]. One animal study showed inhibiting apoptosis of endothelial cells caused by TBI would improve both BBB function and neurological function after TBI [28]. Another two studies found ferroptosis took part in the damage of BMVEC and blood–brain barrier, and inhibition of ferroptosis would reduce BMVEC death, BBB permeability, and tight junction loss after TBI [29, 30]. Additionally, TBI could lead to endothelial dysfunction of systemic terminal vascular bed characterized as impaired endothelial dependent vasodilation through increased arginase activity and endothelial nitric oxide synthase uncoupling with decreased production of nitric oxide [3]. The endothelial dysfunction in the peripheral microcirculation would subsequently promote the adhesion of leukocyte and platelet and the formation of microthrombi with subsequent coagulopathy after TBI manifesting as hyperfibrinolysis and hypercoagulation [3–5]. In our study, the coagulopathy was also confirmed as an independent risk factor for the mortality of TBI, which was similar to previous findings [6, 31, 32]. The coagulopathy is prevalent among TBI patients with the incidence ranging from 7 to 63% [33, 34]. The mortality of TBI patients with coagulopathy ranges from 17 to 86% [33, 35]. Additionally, the coagulopathy has been confirmed associated with progressive hemorrhagic injury and intracranial hemorrhage in TBI patients [36, 37]. In general, more severe vascular endothelial injury may be associated with the poorer prognosis of TBI by reflecting cerebral microvascular injury-induced brain edema and secondary brain injury and peripheral microvascular injury-induced microcirculatory dysfunction and coagulation disorders.

There are several shortcomings in this study. Firstly, TBI patients included into this study derived from a single medical center. The hospital is a regional tertiary medical center mainly treating stubborn and severe disease. The most of TBI patients included were identified as moderate-to-severe TBI as the GCS score shown. Therefore, the selection bias could not be avoided and the conclusion of this study should be verified in more generalized TBI patients from other medical centers. Secondly, levels of endothelial injury markers were not measured so that we could not analyze the true relationship between EASIX and the degree of endothelial injury. The components of EASIX including lactate dehydrogenase, platelet and serum creatinine could be influenced by multiple factors such as bleeding, nutritional status, hepatic and renal function. The mediation effect of endothelial injury on the association between EASIX and prognosis of TBI is not definite. Future studies could be designed to collect levels of endothelial injury markers and testify whether EASIX could be used to reflect the injury severity of vascular endothelium after TBI. Thirdly, only initial EASIX level was measured and analyzed but not the fluctuation of EASIX during hospitalizations. The prognostic value of EASIX change during treatments is worthy to be explored in future studies.

## Conclusion

The EASIX is an effective prognostic marker for TBI patients. The prognostic model incorporating EASIX is helpful for clinicians to evaluate the risk of mortality in TBI patients.

## Data Availability

The datasets used for the current study are available from the corresponding author on reasonable request.
